# How long is the diagnosis of Familial Mediterranean Fever (FMF) delayed in a region where FMF is common in Turkey?

**DOI:** 10.1186/1546-0096-13-S1-P82

**Published:** 2015-09-28

**Authors:** Y Karaaslan, Į Dogan, A Omma, S Can Sandikci

**Affiliations:** 1 Hitit University Medical Faculty, Rheumatology, Çorum, Turkey; 2 Ankara Numune Education and Research Hospital, Rheumatology, Ankara, Turkey; 3 Çorum Education and Research Hospital, Rheumatology, Çorum, Turkey

## Background and question

Similar to other autoinflammatory diseases, diagnosis of FMF is often missed and markedly delayed, particularly when its prevalance is very low in a community. It's reported that diagnosis of FMF might be delayed more than 20 years. Although Turkey is one of the countries with a high prevalence of FMF, the diagnosis of the disease is markedly delayed in clinical practice.

In this study, we aimed to investigate the delay of diagnosis in patients followed up due to, or new diagnosed with FMF in the newly established Rheumatology Unit of a big tertiary medical center located in Çorum region. Çorum province is one of the region where FMF is very common in Turkey.

## Methods

Consecutive 112 patients (38 male, 74 female) who admitted for follow up in the previous 3 months, fulfilling Tel-Hashomer FMF criteria were included in this study.

## Results

The mean age of the patients was 32.9 ± 11.7 years (range 17-59, median 30), mean age at the onset of disease was 13.8 ± 8.8 years (range 2-40, median 10), and the mean age at diagnosis was 25.7 ± 12.8 years (range 3-57, median 23). The mean delay for diagnosis was found as 12 ± 11.8 years (range 0-49, median 8). There was a delay of 10 years or more in 53.6% of the patients, and the delay for diagnosis was 20 years or longer in 11.6% of them.

Among the patients with a delay of diagnosis more than 10 years, before diagnosis of FMF, 11.7 % of the patients were diagnosed with acute rheumatic fever, 5.0% of the patients were diagnosed with rheumatoid arthritis or juvenile chronic arthritis, 8.3% of the patients were diagnosed with spondylarthritis, and 10.0% of the patients were diagnosed with infection and 13.3% of the patients were diagnosed with other disease. 51.6% of the patients did not have any diagnosis.

There was abdominal pain in 92%, fever in 87.5%, arthritis in 35.7%, chest pain in 21.4%,, erysipelas-like rash in 12.5% and history of an increase in acute phase reactants during attack in 97% of our FMF patients. Family history was positive in 65.4% of the patients, and 43.9% of them had history of surgery.

## Conclusions

It is not difficult to diagnose FMF after onset of its clinical symptoms. But the diagnosis of FMF delayed for more than ten years above 50% of patients in Çorum region in Turkey. The most important reason for delay of FMF diagnosis in this region may be lack of “clinical suspicion” for the FMF among primary care and other physicians. Therefore measures must be taken to increase awareness of FMF in physicians and in community in this region.

